# Pivotal Role of GSTO2 in Ferroptotic Neuronal Injury After Intracerebral Hemorrhage

**DOI:** 10.1007/s12031-023-02187-y

**Published:** 2024-02-22

**Authors:** Li Lin, Xiao-Na Li, Zhen-Yan Xie, Yong-Zhen Hu, Qing-Shan Long, Yi-Qi Wen, Xiao-Bing Wei, Li-Yang Zhang, Xue-Song Li

**Affiliations:** 1 Department of Neurosurgery, Huizhou Third People’s Hospital, Guangzhou Medical University, Huizhou, 516002 Guangdong People’s Republic of China; 2 Department of Radiology, the Second Affiliated Hospital, School of Medicine, South China University of Technology, Guangzhou, China

**Keywords:** Hemin, Ferroptosis, Transcriptome analysis, Hippocampal neurons

## Abstract

Previous research has found that an adaptive response to ferroptosis involving glutathione peroxidase 4 (GPX4) is triggered after intracerebral hemorrhage. However, little is known about the mechanisms underlying adaptive responses to ferroptosis. To explore the mechanisms underlying adaptive responses to ferroptosis after intracerebral hemorrhage, we used hemin-treated HT22 cells to mimic brain injury after hemorrhagic stroke in vitro to evaluate the antioxidant enzymes and performed bioinformatics analysis based on the mRNA sequencing data. Further, we determined the expression of GSTO2 in hemin-treated hippocampal neurons and in a mouse model of hippocampus-intracerebral hemorrhage (h-ICH) by using Western blot. After hemin treatment, the antioxidant enzymes GPX4, Nrf2, and glutathione (GSH) were upregulated, suggesting that an adaptive response to ferroptosis was triggered. Furthermore, we performed mRNA sequencing to explore the underlying mechanism, and the results showed that 2234 genes were differentially expressed. Among these, ten genes related to ferroptosis (Acsl1, Ftl1, Gclc, Gclm, Hmox1, Map1lc3b, Slc7a11, Slc40a1, Tfrc, and Slc39a14) were altered after hemin treatment. In addition, analysis of the data retrieved from the GO database for the ten targeted genes showed that 20 items on biological processes, 17 items on cellular components, and 19 items on molecular functions were significantly enriched. Based on the GO data, we performed GSEA and found that the glutathione metabolic process was significantly enriched in the hemin phenotype. Notably, the expression of glutathione S-transferase omega (GSTO2), which is involved in glutathione metabolism, was decreased after hemin treatment, and overexpression of Gsto2 decreased lipid reactive oxygen species level in hemin-exposed HT22 cells. In addition, the expression of GSTO2 was also decreased in a mouse model of hippocampus-intracerebral hemorrhage (h-ICH). The decreased expression of GSTO2 in the glutathione metabolic process may be involved in ferroptotic neuronal injury following hemorrhagic stroke.

**Supplementary Information:**

The online version contains supplementary material available at 10.1007/s12031-023-02187-y.

## Introduction

Hemorrhagic stroke is a severe condition associated with high mortality and disability rates (Keep et al. 2012). Cognitive impairment, particularly in the areas of learning and memory, is one of the most prevalent effects of brain hemorrhage (Delaplain et al. 2020). The hippocampus, a region critical for memory formation, is frequently affected by hemorrhagic stroke (Broadbent et al. 2004). Cognitive impairments following cerebral hemorrhage are typically the result of secondary injury to neurons (Karuppagounder et al. 2016; Zille et al. 2017; Li et al. 2017), which can be induced by a variety of circumstances, including oxidative stress. Hemin (an oxidized form of heme) released from lysed red blood cells (Brott et al. 1997) has been identified as a key contributor to secondary injury in hemorrhagic stroke. Researchers have used hemin as a ferroptosis paradigm to mimic the effects of a hemorrhagic stroke in vitro (Zille et al. 2017). In an in vitro model of cerebral hemorrhage involving hemin, an adaptive transcriptional response involving glutathione peroxidase 4 (GPX4) was induced. However, the induction of GPX4 failed to protect cells from ferroptosis (Alim et al. 2019). Nevertheless, few studies have comprehensively explored the mechanisms underlying frustrated adaptive responses to ferroptosis.

Ferroptosis, a novel iron-dependent form of cell death, is triggered by small compounds (such as erastin, RSL3, and FIN56), lipid peroxide accumulation, glutathione depletion, and hemin exposure (Karuppagounder et al. 2016; Zille et al. 2017; Dixon et al. 2012). In recent years, ferroptosis has been reported as a cause of cell death in cerebral hemorrhage (Li et al. 2017). In addition, cerebral hemorrhage drives a transcriptional response involving GPX4 leading to ferroptosis after the cerebral bleed (Alim et al. 2019). However, the induction of GPX4 in an in vitro model of cerebral hemorrhage involving hemin or in vivo failed to protect against ferroptosis. Notably, the mechanism underlying this failure of the homeostatic transcriptional response to ferroptosis remains unknown.

RNA sequencing (RNA-seq), also known as transcriptome sequencing and particularly mRNA sequencing (mRNA-Seq), can effectively provide comprehensive transcriptional information about organisms under specific physiological conditions (Pang et al. 2017). Thus, RNA sequencing (RNA-seq) was used to compare the genes in the samples, and the differences could provide a scientific basis for studying the pathogenesis of related diseases.

In the present study, we aimed to systematically explore the mechanisms underlying the frustrated adaptive response to neuronal ferroptosis after hemorrhagic stroke in vitro using bioinformatics. To create an in vitro ferroptosis model, we used hemin-treated hippocampal neuronal HT22 cells. Subsequently, we performed mRNA-seq analysis of the hemin-treated and the untreated cells. Based on the mRNA-seq data, we selected target genes associated with ferroptosis and performed GO term analysis. We selected ten gene sets based on the results of GO term analysis and analyzed their functions using gene set enrichment analysis (GSEA). Our findings indicate that glutamate metabolic processes, particularly the Gsto2 gene (related to glutathione metabolism), may play an essential role in ferroptosis neuronal injury. Additionally, we observed a decrease in Gsto2 expression both in vitro and in vivo after hemorrhagic stroke. Our study provides new insights into the frustrated adaptive response to ferroptosis after hemorrhagic stroke and may help identify a potential new therapeutic target for the clinical treatment of hemorrhagic stroke.

## Materials and Methods

### Materials

A live/dead viability/cytotoxicity assay kit for animal cells (catalog number: KGAF001) was obtained from KeyGEN BioTECH. RT-PCR primers were obtained from Sangon Biotech. CELLSAVING (catalog number: C40100), propidium iodide (catalog number: MA0137), and Invitrogen™ BODIPY™ 581/591 C11 (Lipid Peroxidation Sensor) were all purchased from Guangzhou Lead Biotech Biotechnology Company. DFO (catalog number: S6849) was purchased from Selleck. DCFDA-Reactive Oxygen Species Assay Kit (Catalog number: ab113851) was purchased from Abcam. The TRIzol reagent was purchased from Thermo Fisher Scientific. The GSTO2 Antibody (Catalog number: DF12627) was obtained from Affinity. The bicinchoninic acid (BCA) Protein Assay Kit (Catalog number: P0009) was purchased from Beyotime. Monochlorobimane (mBCl, catalog number: 635) was purchased from Zhong Hao Biological.

### Cell Culture of Mouse Hippocampal HT22 Cells and Exposure to Gemin

The mouse hippocampal neurons were incubated in DMEM containing 10% FBS in an incubator at 37 °C and containing 5% CO_2_. When the cell density reached approximately 80%, the cells were exposed to hemin for 6 h. The cell morphology of the untreated hemin was unfurled under an electron microscope, whereas the cell morphology of the treated hemin was reduced. In addition, the cell line in our manuscript has been certified by STR analysis and tested for mycoplasma contamination by FuHeng Cell Center (Shanghai, China). From obiosh.com (Shanghai, China), lentiviruses overexpressing GSTO2 (LV-GSTO2, OE-GSTO2) and empty viruses (LV-NC, Control) were acquired. For a minimum of 5 days, a concentration of 5 ug/mL puromycin was employed to identify positively infected cells.

### Mouse Model of Hippocampus-Intracerebral Hemorrhage

Eight-week-old male C57BJ/L mice were anesthetized by an intraperitoneal injection of 1% sodium pentobarbital (50 mg/kg). After the anesthesia, the skin was prepared, erythromycin eye ointment was applied to the eyes, and the head was sterilized with iodine. The mice were then fixed in a brain stereolocator. A scalpel with an opening of approximately 1 cm was used to make a small incision, and the skull was moistened with normal saline. The brain was stereoscopically positioned with coordinates as follows: 2.5 mm in the front and 1.7 mm in the left (*x* =  − 1.7 mm, *Y* =  − 2.5 mm). The skull was drilled to create a window with a diameter of approximately 1 mm. A Hamilton microsyringe was used to inject 500 nL of 0.045 U/µL collagen-VII-S into the left hippocampus at a rate of 5 nL/s with a downward injection of 1.8 mm (*z* = 1.8 mm). After injection, the needle was retained for 5 min, before slowly being withdrawn. The skin was sutured, and the wound was wiped with iodophor. Finally, 250 µl of normal saline was injected intraperitoneally. The mice were placed on a thermostatic heating pad, allowed to recover, and returned to their cages with access to food and water. Mice in the sham operation group were injected with 500 nL PBS at the same coordinates.

### Viability of the Cells in the Hippocampus Measured by CCK-8

The cell suspension (100 µL/well, 5 × 10^3^ cells/mL) was inoculated into a 96-well plate. Three to five parallel controls were established for each group. The plate was incubated overnight at 37 °C in an incubator containing 5% CO_2_. Before the experiment, the culture solution was removed, and the cells were washed twice with PBS. Subsequently, 10 µL of the CCK-8 solution was added to each well, and the plate was incubated for 2 h. The optical density (OD) at 450 nm was measured. The cell survival rate = (mean OD of experimental group/mean OD of the control group) × 100.

### Propidium Iodide (PI) Staining for Cell Death

HT22 cells were uniformly inoculated into a 6-well plate (at a concentration of 1 × 10^6^ cells/mL). When 70–80% cell fusion was achieved, the cells were treated with hemin for another 6 h. The cells were collected and washed twice with PBS (1000 rpm for 5 min). The cells were re-suspended in 100 µL of PBS, and 2 µL of PI was added to each tube. The process was completed within 30 min using flow cytometry (excitation at 535 nm/emission at 617 nm).

### Lipid Reactive Oxygen Species (ROS), Cytosolic ROS, and GSH Content Analyzed by Flow Cytometry

HT22 cells (2 × 10^5^ cells/well) were seeded in 6-well plates and incubated overnight (37 °C, 5% CO_2_). After treatment with hemin for 6 h according to the experimental design, the cells were collected and resuspended in 500 µL PBS containing 2 µM C11-BODIPY (581/591), 20 µM DCFDA, and 20 µm mBCl. The cells were incubated at 37 °C for 30 min. The fluorescence of each sample was measured using a flow cytometer (excitation at 488 nm/emission at 535 nm or excitation at 394 nm/emission at 490 nm).

### mRNA-Seq Analysis and Gene Enrichment Analysis of HT22 Cells

**Fig. 1:**
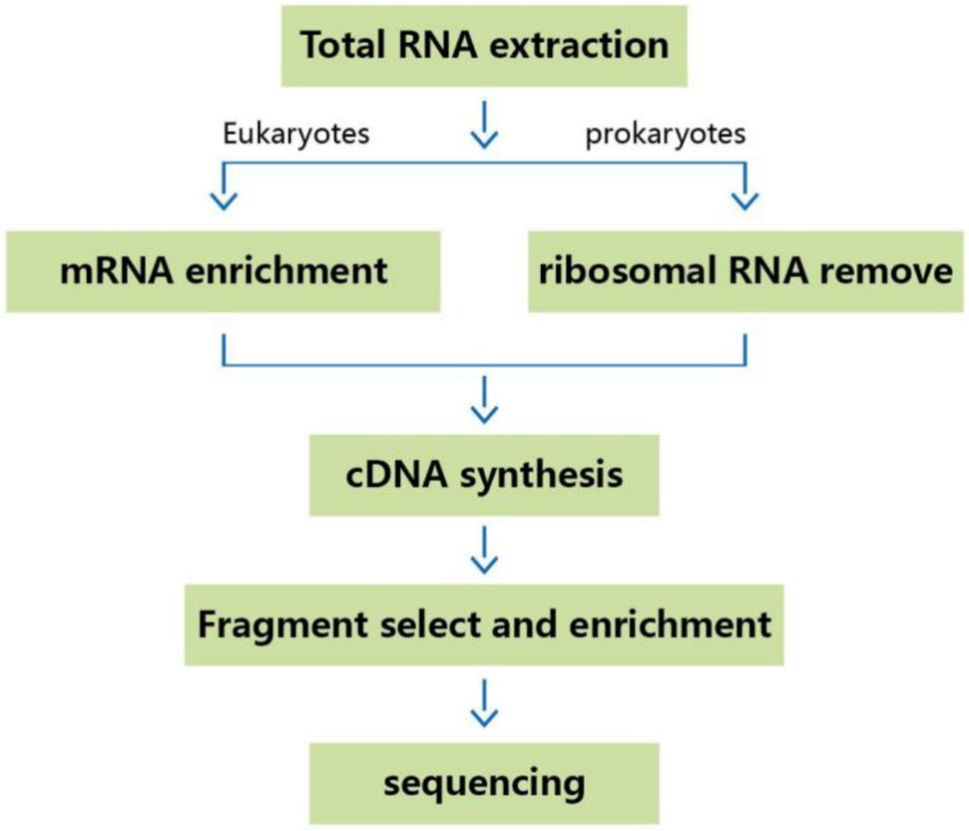
mRNA-Seq experiment process

HT22 cells were harvested after hemin treatment. Total RNA was extracted using a TRIzol reagent according to the manufacturer’s protocol. RNA purity and quantity were evaluated using a NanoDrop 2000 spectrophotometer (Thermo Fisher Scientific, USA). RNA integrity was assessed using an Agilent 2100 Bioanalyzer (Agilent Technologies). Libraries were constructed using the TruSeq Stranded mRNA LT Sample Prep Kit (Illumina, San Diego, CA, USA) according to the manufacturer’s instructions (Fig. 1). Transcriptome sequencing and analysis were performed by OE Biotech Co., Ltd. (Shanghai, China).

The libraries were sequenced on an Illumina HiSeq × Ten platform, and 150 bp paired-end reads were generated. Raw data were initially processed using Trimmomatic (Kim et al. 2015), and low-quality reads were removed to obtain clean reads. Clean reads were mapped to the mouse genome (GRCm38.p6) using HISAT2. The fragments per kilobase of transcript per million mapped reads (FPKM) of each gene were calculated using Cufflinks, and the read counts of each gene were obtained using HTSeq-count. Differential expression analysis was performed using the DESeq R package (2012). A *P*-value of < 0.05 and a fold change of > 2 or < 0.5 were set as the thresholds for significantly differential expression. A hierarchical cluster analysis of the differentially expressed genes (DEGs) was performed. GO enrichment and KEGG pathway enrichment analysis of the DEGs were performed using R based on the hypergeometric distribution.

### Real-time Quantitative PCR Analysis

Total RNA was isolated from cell samples according to a previous protocol (Chen et al. 2018). The RNA was then reverse-transcribed into cDNA using the PrimeScript™ RT reagent Kit (Takara, Catalog number: RR047A). mRNA primers were obtained from Sangon Biotech. mRNA expression levels of GPX4, SLC7A11, SLC3A2, and Gsto2 (Table 1) were detected using a Real-Time System (Bio-Rad). The genes of GPX4, SLC7A11, Gsto2, and SLC3A2 were relativized to β-actin.

**Table 1:** RT-PCR primers

Genes	Genes name	Sequence (5′ → 3′)
*GPX4*	GPX4-F	ATAAGAACGGCTGCGTGGTGAAG
	GPX4-R	TAGAGATAGCACGGCAGGTCCTTC
*SLC7A11*	SLC7A11-F	TGGATGCTGTGCTTGGTCTTGATG
	SLC7A11- R	CTGCCTGCTGTACCGTGGTTATG
*SLC3A2*	SLC3A2-F	GGTGGTGCTCAACTTCCGAGATTC
	SLC3A2-R	CGCTGGCTGGCAGGCTTATG
*Gsto2*	Gsto2-F Gsto2-R	TGTGGTCTCCTCGGCGGTTATC AGACGGACAGGCTGGATGGTAAG

### Western Blotting for the Expression of GSTO2

HT22 cells and the hippocampus were harvested using cell lysates, and the total protein concentration was determined using the bicinchoninic acid (BCA) Protein Assay Kit. Proteins in each group were diluted with five times the loading buffers to the same concentration, and equivalent amounts of proteins were subjected to sodium dodecyl sulfate–polyacrylamide gel electrophoresis. After electrophoresis, the proteins were transferred to a PVDF membrane using a wet transfer system and sealed with TBST (0.1% Tween-20, 150 mM NaCl, and 50 mM Tris-HCl, pH 7.5) at room temperature. After 2 h, the membranes were rinsed three times (10 min/time) with TBST and incubated with the GSTO2 antibody (1:1000) at 4 °C in a shaker overnight. The following day, the membranes were rinsed as described above and incubated with an anti-rabbit antibody (1:5000) for 2 h. Finally, the membranes were washed with TBST (three times, 10 min/wash), and protein expression was detected using an enhanced chemiluminescence imaging system. The gray value was scanned using ImageJ software.

### Hemorrhage Volume Analysis

Mice were put under anesthesia, and their brains were harvested 24 h following h-ICH. The 1-mm brain slices were prepared and fixed for 1 min in a 4% paraformaldehyde (PFA) solution. The volume of the hemorrhage was assessed using digital imaging, and the photographs were processed using ImageJ software.

### Statistical Analysis

All data are presented as mean ± standard error (SEM). GraphPad Prism 8 was used for statistical analyses. One-way ANOVA was used for multiple-group comparisons, and an unpaired *t*-test was used for two-group comparisons. Each experiment was repeated three or more times. ^**^P < 0.01, ^***^P < 0.001, and ^*^P < 0.05 were considered statistically significant.

## Results

### Toxic Effects of Hemin on Hippocampal Neuronal HT22 Cells

**Fig. 2:**
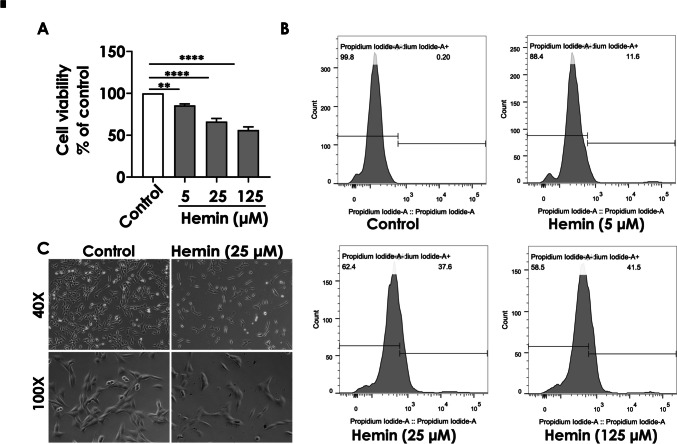
Toxic effects of hemin on hippocampal neuronal HT22 cells. The HT22 cells were treated with hemin (25 µM) for 6 h. **A** Cell viability was determined using CCK-8. **B** Cell mortality determined using PI staining (fluorescence profiles and mean fluorescence intensity (MFI)). **C** Representative images of the live HT22 cells (Calcein-AM, green) exposed to hemin (25 µM); scale bars represent a magnification of 100**× **. The values are presented as the mean ± S.E.M., *n* = 3. ^*^P < 0.05, ^**^P < 0.01, ^****^P < 0.0001, versus the control group

Ferroptosis is a recently identified type of cell death that differs from apoptosis, necrosis, and autophagy. It has been seen in the neurological system, renal tissue, and cancer cells (Dixon et al. 2012). We focused on hemin-induced ferroptosis in HT22 hippocampal neuronal cells to examine the effects of ferroptosis on brain injury after hemorrhagic stroke in vitro*.* To investigate the harmful effects of hemin on the HT22 cells, we first used a CCK-8 kit and flow cytometry to measure cell viability and mortality, respectively. After the exposure to different concentrations (5, 25, and 125 µM) of hemin for 6 h, the viability of the HT22 cells decreased (Fig. 2A, P < 0.01, *P* < 0.0001, respectively), and cell mortality increased with increasing concentrations of hemin (Fig. 2B). The HT22 cells were treated with hemin for 6, 12, and 24 h, and cell viability was approximately 10% at 12 h (Supplementary Fig. 1). In addition, the results showed that cell mortality reached nearly 40% at a concentration of 25 µM after treatment with hemin for 6 h (Fig. 2B), which was used as an appropriate concentration for further studies of ferroptosis mechanisms. At this concentration, ferroptosis occurred without excessive cell death. To observe the viability of HT22 cells more accurately after hemin treatment, we used calcein-AM to label living cells, and the cell viability was observed using laser confocal microscopy. As shown in Fig. 2C, the number of live HT22 cells decreased after hemin treatment, consistent with the quantitative results presented in Fig. 2A and B.

### Hemin Triggers Lipid Peroxide Accumulation and an Adaptive Response to Ferroptosis in HT22 Hippocampal Neuron Cells

**Fig. 3:**
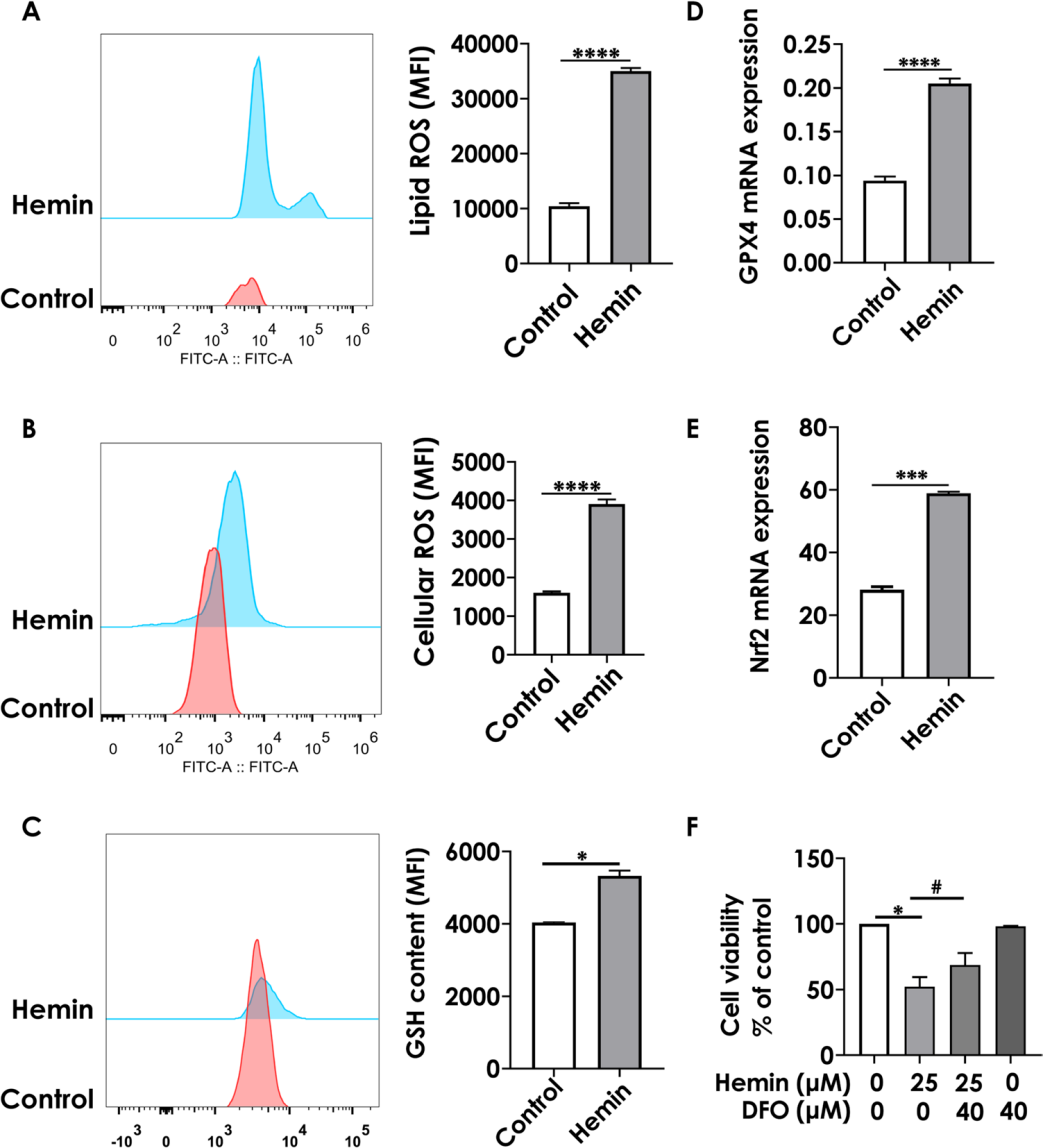
Effects of hemin on lipid peroxides and ferroptosis-related genes in HT22 hippocampal neuron cells. HT22 cells exposed to hemin (25 µM) for 6 h. **A** and **B** lipid ROS and cellular ROS measured using flow cytometry after C11-BODIPY staining and DCFDA staining, respectively. **C** GSH content was detected using flow cytometry. **D** and **E** mRNA expressions of GPX4 and Nrf2 measured using quantitative real-time polymerase chain reaction (PCR). **F** Cell viability determined using CCK-8. The values are presented as mean ± S.E.M., *n* = 3. ^*^P < 0.05, ^***^P < 0.001, ^****^P < 0.0001, versus the control group; ^#^*P* < 0.05, versus the hemin-treated group

To find new methods for reversing hemin-induced toxicity, we concentrated on ferroptosis in neurons, a kind of cell death that is dependent on intracellular iron levels and the formation of lipid peroxides (Gaschler and Stockwell 2017). Additionally, ferroptosis can be prevented by using iron chelators (Dixon et al. 2012). In our study, exposure to hemin (25 µM, for 6 h) significantly increased the lipid ROS and cytosolic ROS content in HT22 cells (Fig. 3A and B). However, pretreating the cells with an iron chelator (DFO) resulted in a significant increase of nearly 25% in the viability of hemin-exposed HT22 cells (Fig. 3F, P < 0.05).

To further explore the mechanism underlying the frustrated adaptive response to ferroptosis, we probed changes in the mRNA levels of antioxidant enzymes, such as GPX4, glutathione (GSH), and nuclear factor E2 related factor 2 (Nrf2). The GPX4 neutralizes reactive lipids during ferroptosis (Forcina and Dixon 2019), whereas the GSH, a synthetic substrate of GPX4, is required for its lipid neutralization function (Feng and Stockwell 2018). Nrf2, an antioxidant transcription factor, regulates hundreds of antioxidant genes, including GPX4 (Wu et al. 2011). Consistent with the finding of previous research (Alim et al. 2019), the mRNA expression of GPX4 (Fig. 3D, P < 0.0001), GSH (Fig. 3C, P < 0.001), and Nrf2 (Fig. 3E, P < 0.001) was increased in hemin-treated HT22 cells. Collectively, these results suggest that an adaptive response to ferroptosis is triggered after a hemorrhagic stroke in vitro. However, the adaptive response failed to protect HT22 cells from ferroptosis. Based on the established model, we performed transcriptome sequencing for a holistic analysis.

### Analysis of Transcriptome Sequencing Database After Hemin Exposure

**Fig. 4:**
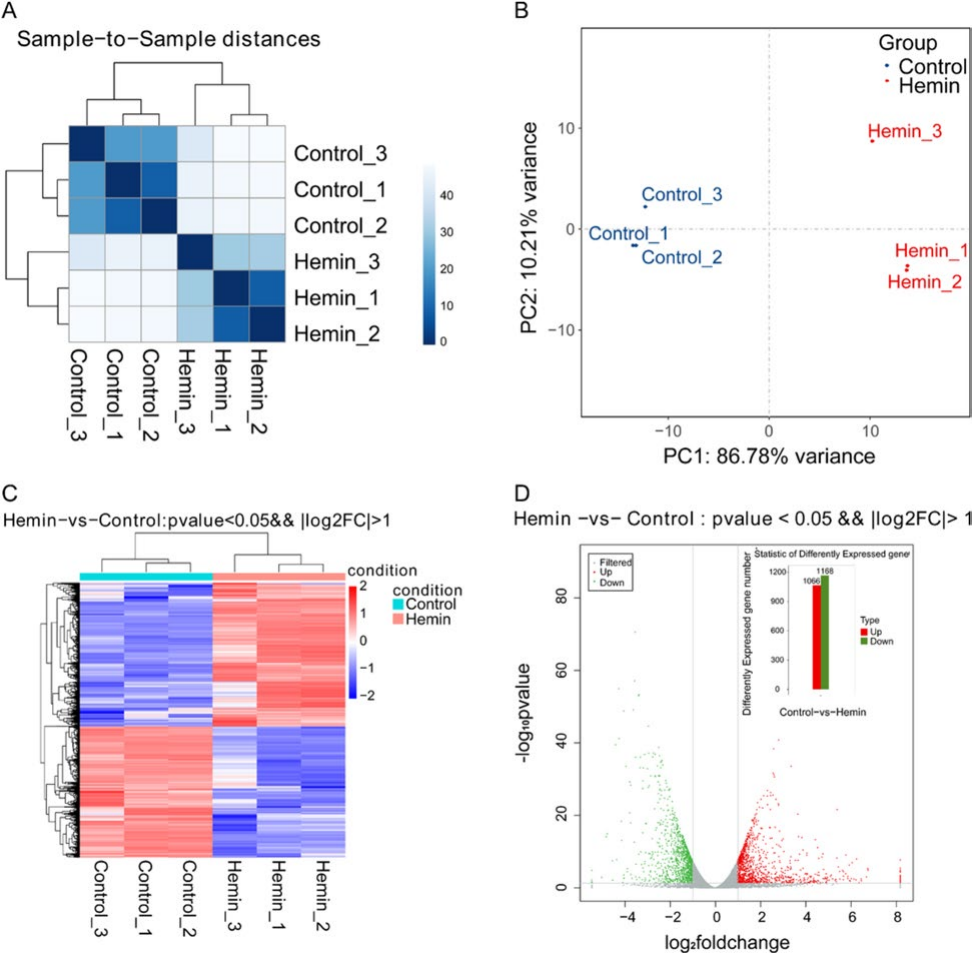
Analysis of gene expression profiles after the exposure of HT22 cells to hemin. **A** Results of cluster analysis. The X-coordinate represents the sample name, the Y-coordinate represents the corresponding sample name, and the color represents the correlation coefficient. **B** Results of principal component analysis (PCA). **C** Heatmaps of mRNA expressions in the hemin-treated and control groups. Red represents highly expressed RNAs and blue represents mRNAs with low expression. **D** The volcano plot and statistic of differentially expressed genes (DEGs) in the control and hemin-treated groups. Gray dots represent RNAs that are not significantly different, and red (upregulated mRNAs) and green (downregulated mRNAs) dots represent RNAs that are significantly different

To thoroughly investigate the mechanism underlying hemin-induced ferroptosis, we analyzed the relationship between the two experimental groups of HT22 cells (with and without hemin) using transcriptome sequencing. Cluster analysis was conducted to examine the similarities between the samples (Fig. 4A). Principal component analysis (PCA) was used to explore the intersample relationships between the two groups (Fig. 4B). In the PCA plot, the expression profiles of the samples exposed to hemin were separated from those of the control group (without treatment).

In addition, differential gene expression analysis (DEG) was performed to identify changes in gene expression between the hemin-treated and control groups (Fig. 4C and D). The gene expression pattern of the hemin-treated group was found to be opposite to that of the control group. As shown in the heatmap (Fig. 4C) and volcano (Fig. 4D) results, a total of 2234 DEGs (∣log2FC∣ > 1 and *P* < 0.05) were detected in the hemin-treated and control groups, of which 1066 genes were upregulated (upper half of the heatmap) and 1168 genes were downregulated (lower half of the heatmap) after hemin treatment.

### Identification of the Target DEGs Associated with Ferroptosis

**Fig. 5:**
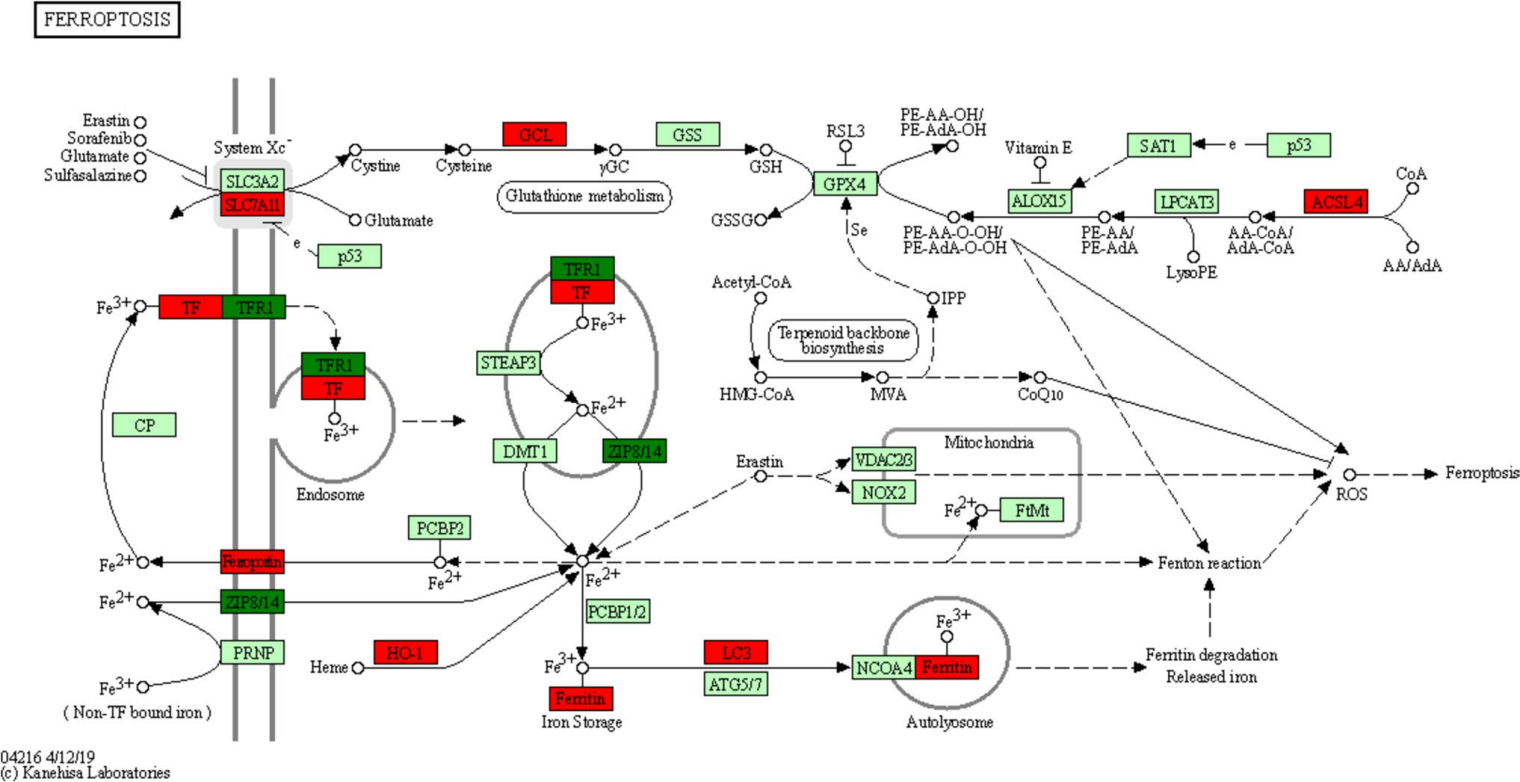
KEGG network of ferroptosis pathway based on RNA sequence. The genes in red (Acsl1, Ftl1, Gclc, Gclm, Hmox1, Map1lc3b, Slc7a11, Slc40a1) represent upregulated genes, and the genes in green (Slc39a14, Tfrc) represent downregulated genes after hemin treatment

**Fig. 6:**
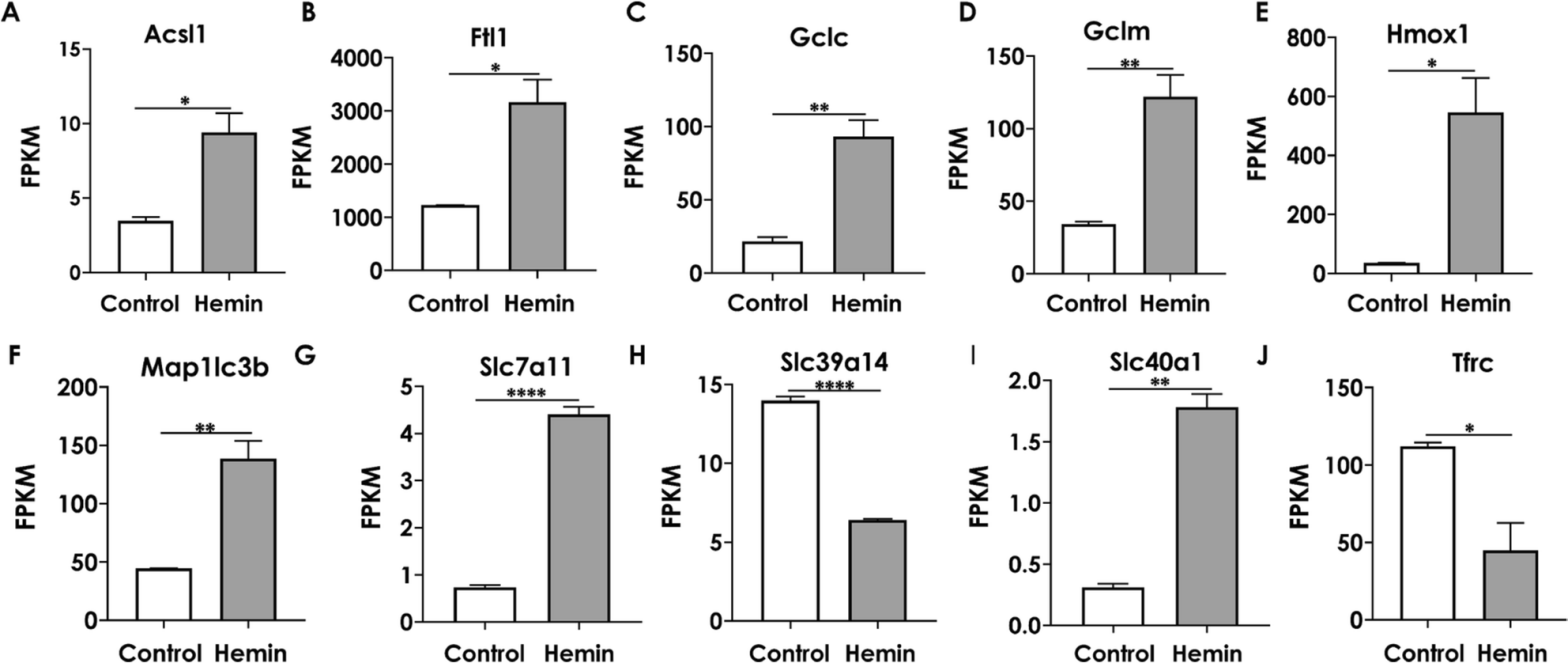
Changes in gene levels involved in the ferroptosis pathway after hemin exposure (25 µM, 6 h). **A**–**J** Relative mRNA levels of ferroptosis-related key genes associated with KEGG pathway “ferroptosis.” The values are presented as mean ± S.E.M., *n* = 3. ^*^P < 0.05, ^**^P < 0.01, ^****^P < 0.0001, versus control group

To better understand the genes involved in hemin-induced ferroptosis and their significant differences, the ten differentially expressed mRNAs are presented in Supplementary Table 1 based on the KEGG network of the ferroptosis pathway (Fig. 5). Among them, most genes (Acsl1, Ftl1, Gclc, Gclm, Hmox1, Map1lc3b, Slc7a11, and Slc40a1) were upregulated, whereas Tfrc and Slc39a14 were downregulated. The expression levels of these ten genes are depicted in statistical graphs in Fig. 6A–J.

### Analysis of the Functional Enrichment of Ten Targeted DEGs Involved in Ferroptosis

**Fig. 7:**
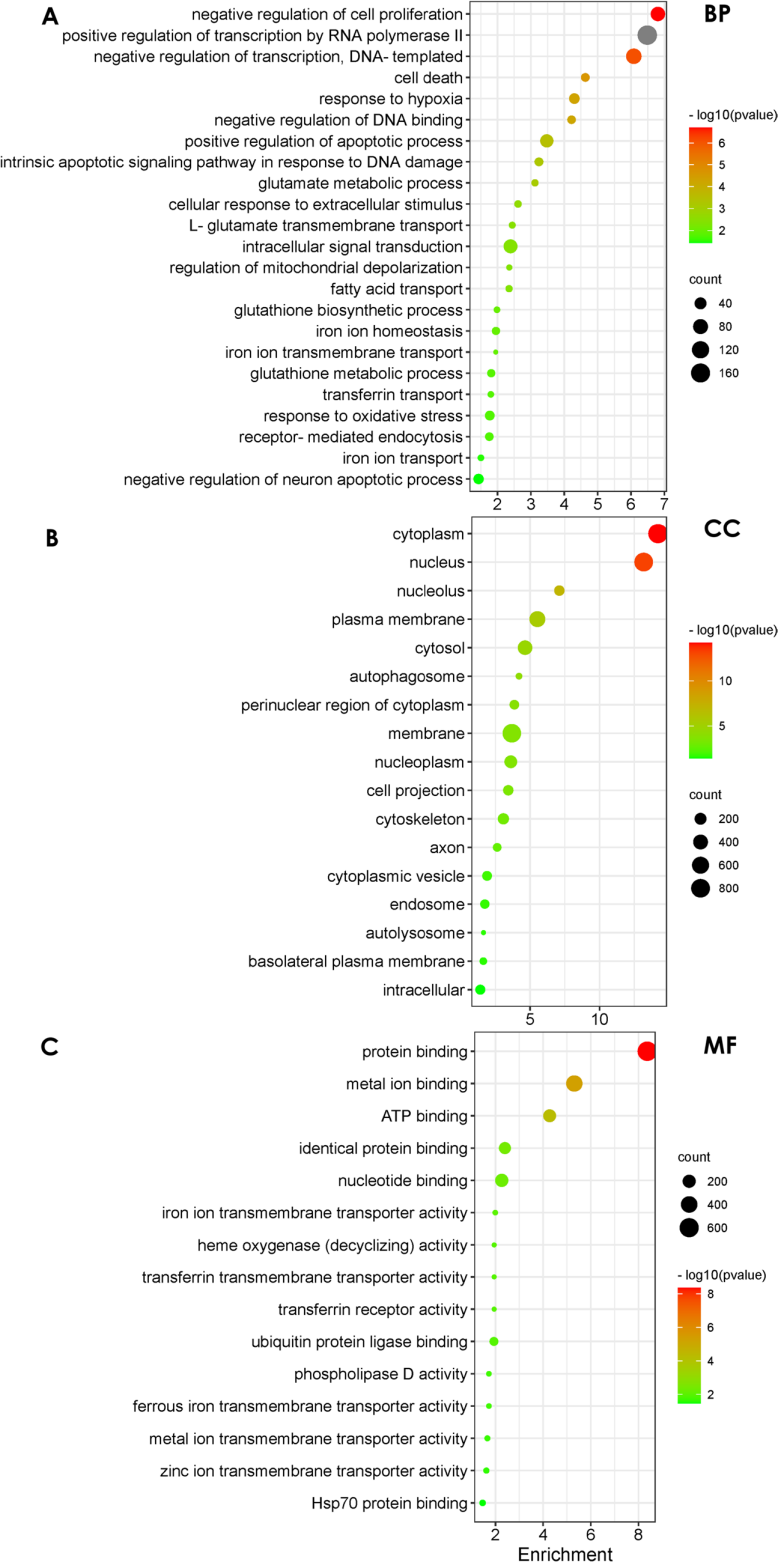
Functional enrichment analysis of DEGs. GO term enrichment analysis of DEGs for the ten target genes involved in the ferroptosis pathway. **A** BP represents biological process; **B** CC represents cellular component; **C** MF represents molecular function

To gain a better understanding of the ten targeted DEGs associated with ferroptosis, we performed a GO analysis of those ten genes (Fig. 7). The functional enrichment results revealed 20 significantly enriched terms for biological processes (BP), and negative regulation of cell proliferation, positive regulation of transcription by RNA polymerase II, negative regulation of transcription, and DNA-templated cell death were the most enriched BP terms (Fig. 7A). Additionally, 17 cellular components (CC) items, such as the cytoplasm, autophagosome, and cytoskeleton organization, were significantly enriched, with the cytoplasm being the most enriched CC term (Fig. 7B). Moreover, 19 molecular function (MF) items, including protein binding, metal ion binding, HSP70 protein binding, and regulation of ubiquitin-protein ligase activity, were significantly enriched, with protein binding, metal ion binding, and ATP binding being the most enriched MF terms (Fig. 7C).

### Gene Set Enrichment Analysis (GSEA) Against the Gene Sets of the GO Database

**Fig. 8:**
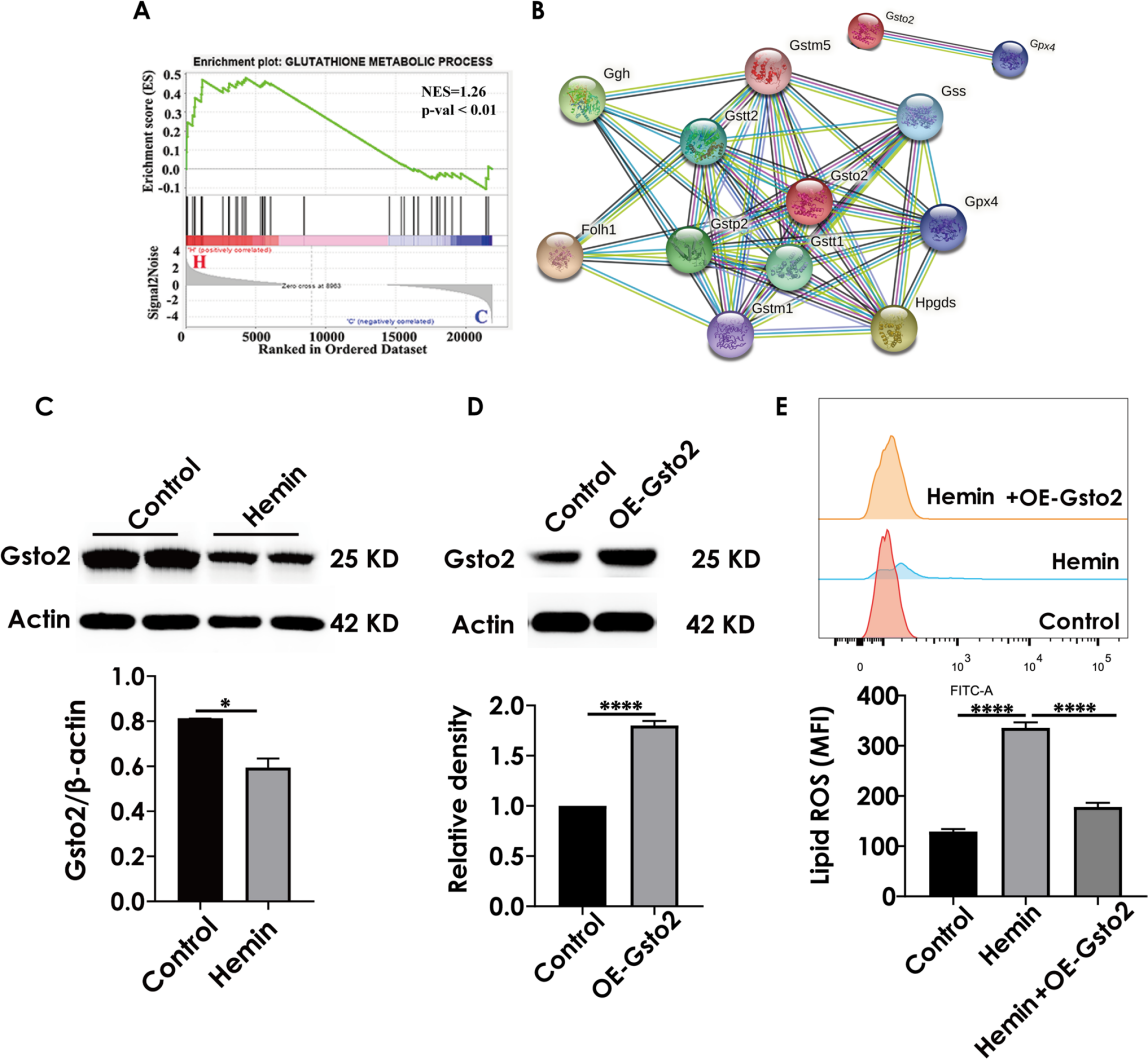
GSEA against the obtained GO database in hemin-treated HT22 cells and the expression of Gsto2 was detected by western blot. **A** GSEA plots of the gene sets upon the GO database after hemin treatment. **B** String PPI analysis of the interaction network of Gsto2. **C**, **D** Expression of Gsto2 detected by western blotting. **E** Level of lipid ROS detected by flow cytometry

To holistically analyze the effect of hemin on cell function, we performed gene set enrichment analysis (GSEA) based on the gene sets identified in the GO enrichment analysis (Fig. 7). We found that the “glutathione metabolic process” was enriched in the hemin phenotype (Fig. 8A). The glutathione metabolic process was closely associated with ferroptosis (Gao et al. 2015). Notably, Gsto2 (glutathione S-transferase omega 2) is involved in the glutathione metabolic process as an antioxidant enzyme that has a protective role in neuronal cells (Allen et al. 2012). Using the String online tool, we identified ten Gsto2 binding proteins, including GPX4 (Fig. 8B). It has been suggested that Gsto2 has the dual functions of scavenging peroxides and detoxification by catalyzing the binding of GSH to harmful substances (Kim et al. 2017). GSH is the key cosubstrate of GPX4, and the GSH-GPX4 pathway acts as a key regulator in the development of ferroptosis (Friedmann Angeli et al. 2014; Yang et al. 2014). In addition, we detected the expression of Gsto2 by western blotting and found that Gsto2 expression decreased in hemin-treated HT22 cells (Fig. 8C). To confirm that Gsto2 was involved in ferroptotic neuronal damage, we used flow cytometry to detect the lipid ROS level after manipulating Gsto2 expression. As shown in Fig. 8D and E, overexpression of Gsto2 led to a decreased lipid ROS level in hemin-exposed HT22 cells. These results indicated that Gsto2 may play a vital role after intracerebral hemorrhage.

### Hemorrhage Volume in the Brain Increased and the Expression of Gsto2 Was Decreased After the Hemorrhagic Stroke

**Fig. 9:**
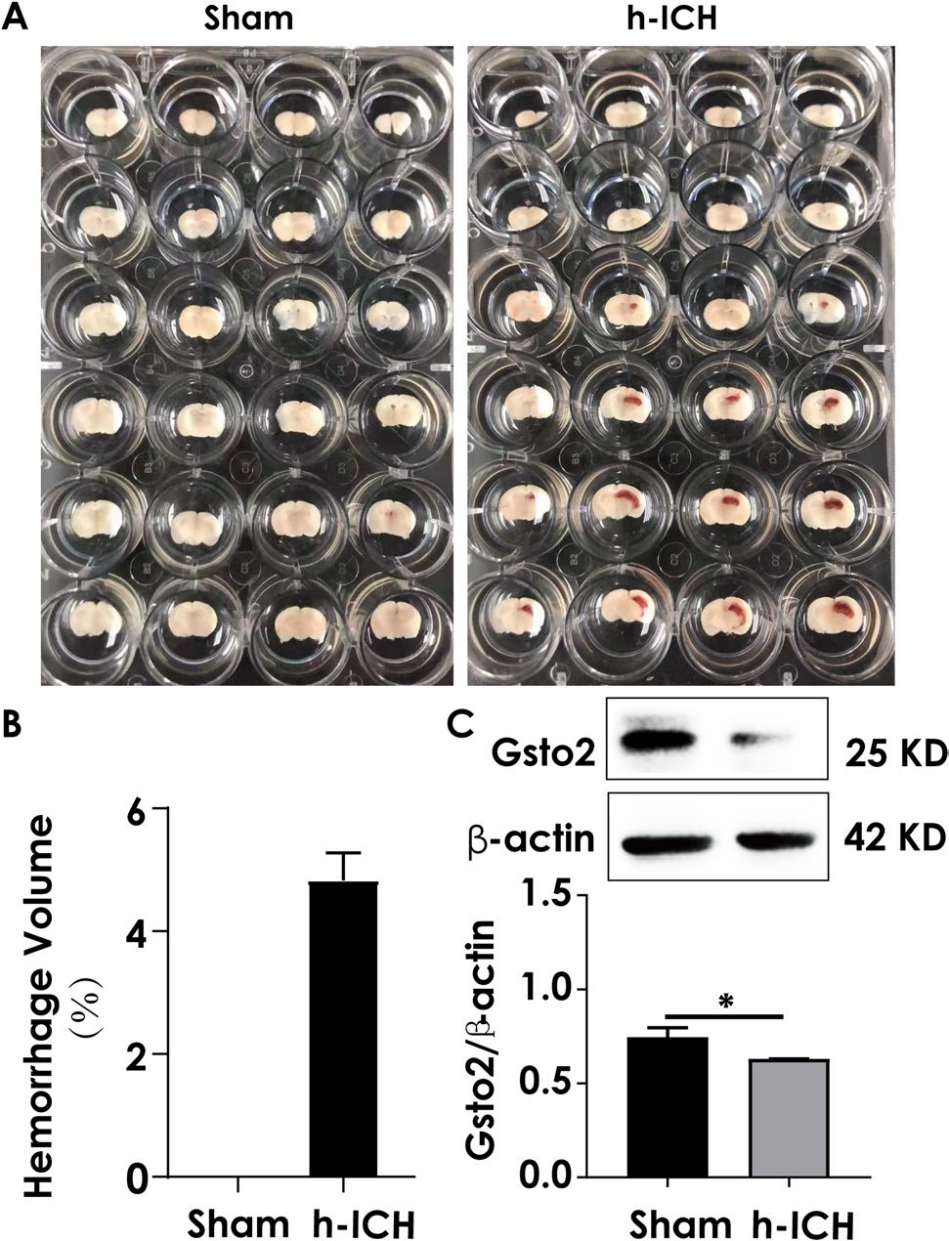
Hemorrhage volume following hemorrhagic stroke and the expression of Gsto2 in the hippocampus. **A** Representative images of brain sections at 24 h post-injury. **B** Hemorrhage volume measured in the brain (*n* = 4). **C** Expression of Gsto2 in the hippocampus detected by western blotting (*n* = 4)

To investigate whether Gsto2 is involved in ferroptotic neuronal damage after hemorrhagic stroke, we established a hippocampal hemorrhage mouse model (h-ICH; Fig. 9A and B). As shown in Fig. 9C, Gsto2 expression decreased in the hippocampus 24 h post-injury. This suggests that Gsto2 plays a pivotal role in ferroptotic neuronal injury following hemorrhagic stroke.

## Discussion

Hemorrhagic stroke, which occurs when a blood artery ruptures in the brain, is a common kind of stroke with a high mortality and morbidity rate (Montaño et al.  2021). Early identification and treatment are essential because bleeding that is expanding quickly can cause a dramatic decline in cognitive abilities, including neurological dysfunction and loss of consciousness. Therefore, it is imperative to thoroughly investigate the pathophysiology of stroke and create efficient diagnostic and therapeutic approaches. After cerebral hemorrhage, ferroptosis develops and contributes to neuronal death (Bai et al. 2020). Following cerebral hemorrhage, an adaptive transcriptional response involving GPX4 is induced after cerebral hemorrhage. However, it fails to protect cells against ferroptosis (Alim et al. 2019). In addition, Hb/heme/iron is also thought to be one of the main causes of delayed cerebral edema and irreversible neuronal injury, and it is crucial for the generation of lipid reactive oxygen species (ROS) following hemorrhage (Xiong et al. 2014). Hemin causes ferroptosis after intracerebral hemorrhage (ICH), according to a recent investigation (Derry et al. 2020). As a result, developing effective medications and treatments for post-stroke nerve injury requires a thorough knowledge of the mechanism underlying this failed transcriptional response to ferroptosis. However, the molecular processes causing the lack of transcriptional responses to ferroptosis remain unknown and need to be investigated further.

RNA-Seq analysis based on next-generation sequencing (NGS) is an evolving modern technique for transcriptome profiling (Sudhagar et al. 2018). mRNA, a single-stranded RNA transcribed from a strand of DNA used as a template, carries genetic information that guides protein synthesis. Therefore, mRNA-based transcriptome analysis has become the best tool for revealing the mechanism underlying the occurrence and development of diseases and identifying key targets for regulating pathogenic genes (Mutz et al. 2013). Moreover, this rapid and effective genomic survey method could be used to identify large-scale functional genes and molecular markers (Morozova et al. 2009). In the present study, we systematically analyzed the role of mRNA in hemin-induced ferroptosis and explored the underlying mechanism.

First, we detected and analyzed 2234 differential genes before and after hemin treatment in HT22 cells, 1168 genes were upregulated and 1066 genes were downregulated. To better investigate the mechanisms underlying ferroptosis, ten target genes involved in ferroptosis were identified based on the ferroptosis KEGG signalling pathway. These genes were Acsl1, Ftl1, Gclc, Gclm, Hmox1, Map1lc3b, Slc7a11, and Slc40a1, which were upregulated. For the ten ferroptosis-associated genes, we performed the GO ontology analysis and found that the differentially expressed genes were enriched in fatty acid transport, cytoskeleton, autophagosome, and transferrin transport. Furthermore, ferroptosis mainly occurs through the accumulation of polyunsaturated fatty acids (PUFA) in the cell membrane, because PUFA determines the degree of lipid peroxidation in cells (Yang and Stockwell 2016). The PUFA and cell membranes regulate the cytoskeleton (Schmidt et al. 2015). Additionally, ferritin, the main iron storage, plays an important role in preventing iron overload and can be selectively degraded through selective autophagy, namely ferritinophagy (Bellelli et al. 2016). These findings are consistent with the genes enriched in fatty acid transport pathways, cytoskeleton, and autophagosomes after hemin treatment. Thus, GO function enrichment provides valuable research directions and ideas for studying the mechanism underlying ferroptosis caused by hemin. We performed GSEA to explore the effect of hemin on cell function from a holistic perspective. According to GSEA, the glutathione metabolic process was significantly enriched in HT22 cells incubated with hemin. glutathione is a vital regulator of ferroptosis and its absence induced the formation of GPX4 (Stockwell et al. 2017). By constructing the PPI network of Gsto2 in the glutathione metabolic process, we found that GPX4 plays a central role in the network.

Gsto2 (glutathione S-transferase omega 2, EC2.5.1.18) is a multifunctional isoenzyme that is widely distributed in various organisms. Notably, GSTO2 facilitates the reduction of glutathione (GSH). More importantly, the glutathione (GSH)-glutathione peroxidase-4 (GPX-4) pathway is key to regulating ferroptosis (Gaschler and Stockwell 2017; Friedmann Angeli et al. 2014), and the depletion of GSH can induce ferroptosis (Dixon et al. 2012; Yang et al. 2014). Overexpression of Gsto2 induced decreases in lipid ROS level of hemin-exposed HT22 cells. To confirm the alteration of GSTO2, we detected GSTO2 protein levels by western blotting. The expression of GSTO2 protein decreased after hemin treatment in a mouse model of hippocampus-intracerebral hemorrhage, indicating that GSTO2 plays an important role in the adaptive response to ferroptosis. These findings provide valuable research directions and molecular targets for subsequent mechanistic studies. 

## Conclusions

In summary, our study presents the first macroscopic analysis of the mRNA-based transcriptome sequencing of HT22 cells treated with hemin. The results demonstrated the considerable involvement of Gsto2 in the glutathione metabolic processes. These findings enhance the current understanding of the mechanisms underlying the frustrated adaptive responses to ferroptosis and provide a series of goals and hypotheses to guide future research.

### Supplementary Information

Below is the link to the electronic supplementary material.

Supplementary file1 (DOCX 83 KB)

## Data Availability

The data and materials used to support the findings of this study are available from the corresponding author upon request. The sequencing data contained in my manuscript has been successfully deposited in the Sequence Read Archive (SRA). The accession numbers are respectively SRR16095410, SRR16095411, SRR16095412, SRR16095413, SRR16095414, and SRR16095415. This is the link to sequencing data in SRA: https://dataview.ncbi.nlm.nih.gov/object/PRJNA765144?reviewer=gsgbf3tnq3qtq2lvcqfktru597.

## Abbreviations

Slc7a11: Solute carrier family 7 member 11
Ftl1: Ferritin light chain 1
Gclc: Glutamate-cysteine ligase catalytic subunit
Gclm: Glutamate-cysteine ligase modifier subunit
Hmox1: Heme oxygenase 1
Slc39a14: Solute carrier family 39 member 14
Slc40a1: Solute carrier family 40 member 1
Acsl1: Acyl-CoA synthetase long-chain family member 1
Map1lc3b: Microtubule-associated protein 1 light chain 3 beta
Tfrc: Transferrin receptor
KEGG: Kyoto Encyclopedia of Genes and Genomes
