# Supplementary material for: Pivotal Role of GSTO2 in Ferroptotic Neuronal Injury After Intracerebral Hemorrhage
Source: J Mol Neurosci. 2024 Feb 22;74(1):24. doi: 10.1007/s12031-023-02187-y (PMC10884062; doi:10.1007/s12031-023-02187-y)
Supplement: Supplementary file 1 — Supplementary file1 (DOCX 83 KB) [file 12031_2023_2187_MOESM1_ESM.docx]

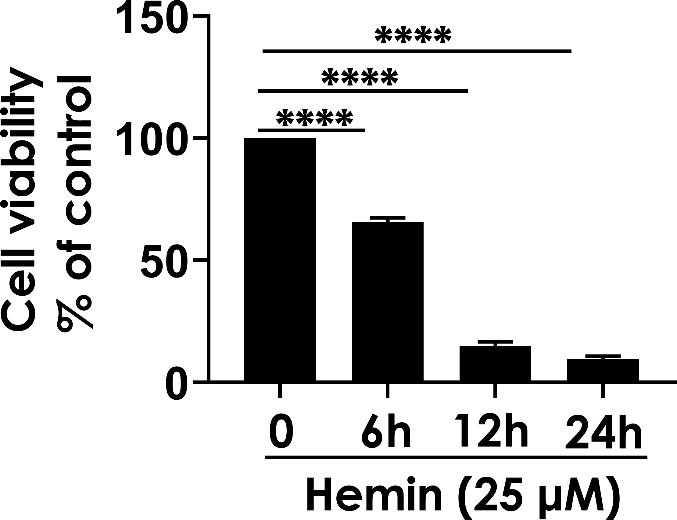


**Supplementary Figure 1.** HT22 cells were treated with hemin (25 μM) for 6 h, 12 h, 24 h. The cell viability was detected by CCK-8. Values are the mean ± S.E.M., n=3. ^****^*P* < 0.0001, versus control group.

**Supplementary Table 1. Differentially expressed mRNAs involved in ferroptosis after hemin treatment of HT22 hippocampal neuronal cells**

**
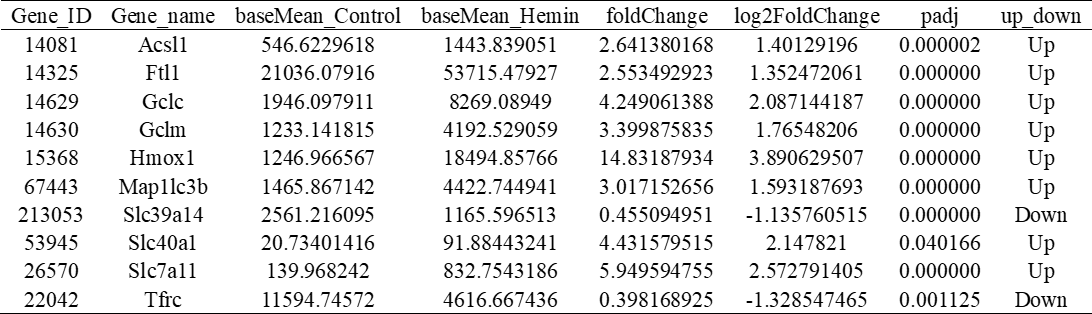
**
